# Impact of continuous local anesthetic wound infiltration on postoperative outcomes following open radical cystectomy: a comprehensive evaluation

**DOI:** 10.1007/s00345-026-06419-9

**Published:** 2026-04-19

**Authors:** François Crettenand, Alkiviadis Papagiannakis, Kevin Stritt, Nuno Grillo, Beat Roth, Sonia Rodrigues Dias, Jean-Daniel Rouvé, Catherine Blanc, Ilaria Lucca

**Affiliations:** 1 Department of Urology, Lausanne University Hospital and University of Lausanne, Rue du Bugnon 46, Lausanne, 1011 Switzerland; 2 Department of Anesthesiology, Lausanne University Hospital, University of Lausanne, Lausanne, 1011 Switzerland; 3 Department of Urology, Bern University Hospital, University of Bern, Bern, Switzerland; 4 Department of Urology, KSA Kantonspital Aarau, Aarau, Switzerland

**Keywords:** Cystectomy, Bladder cancer, Pain management, Postoperative care, Enhanced recovery after surgery

## Abstract

**Purpose:**

To evaluate continuous wound infiltration (CWI) as an alternative to thoracic epidural analgesia (TEA) for postoperative recovery after open radical cystectomy within an ERAS^®^ pathway.

**Materials and methods:**

Retrospective before–after cohort at a Swiss tertiary center using a prospectively maintained ERAS^®^ database (TEA: Nov 2012–May 2016; CWI: Jun 2016–Mar 2024). Adults undergoing open cystectomy with extended pelvic lymphadenectomy and ileal conduit or neobladder were included (CWI = 154; TEA = 88). Outcomes were daily pain (VAS 0–10), nausea, opioid rescue (POD0–3), gastrointestinal recovery (first stool; tolerance of solid food), length of stay (LOS), and 90-day complications. Mixed-effects and multivariable models adjusted for prespecified covariates.

**Results:**

Pain scores were lower with TEA on POD0–3, whereas CWI had less postoperative nausea. CWI showed faster bowel recovery (first stool median 5 vs. 6 days, *p* = 0.02) and earlier tolerance of solid food (median 5 vs. 11 days, *p* < 0.001; adjusted β − 4.4 days, 95% CI − 6.8 to − 2.1). LOS was shorter with CWI (median 15 vs. 24 days, *p* < 0.001; adjusted β − 5.0 days, 95% CI − 9.1 to − 1.0). Early opioid rescue was more frequent with CWI, attenuating by POD3. No CWI catheter-related complications occurred.

**Conclusion:**

Within ERAS^®^, CWI with ropivacaine is a clinically acceptable alternative to TEA after open RC, trading slightly higher early pain for faster gastrointestinal recovery and a shorter LOS.

## Introduction

Radical cystectomy (RC) with extended pelvic lymphadenectomy (PLND) and urinary diversion, remains the gold-standard treatment for muscle-invasive bladder cancer and selected high-risk non-muscle invasive cancer [1]. Despite the increasing adoption of robot-assisted radical cystectomy, contemporary randomized and high-level comparative data indicate broadly similar short-term complication profiles versus open RC. have reported similar complication rates [2–4].

Enhanced Recovery After Surgery (ERAS^®^) pathways introduced for RC more than a decade ago, have led to a significant reduction in length of stay (LOS) and morbidity through multimodal optimization [5, 6]. A key component of ERAS^®^ protocols is the postoperative pain management. Thoracic epidural analgesia (TEA) has long provided excellent pain relief and can facilitate early mobilization within multimodal care. However, neuraxial techniques require expertise and monitoring, and can be associated with hypotension, urinary retention and rare neurological complications [7] Continuous wound infiltration (CWI) with local anesthetic via a preperitoneal catheter is an appealing, simpler alternative that may improve gastrointestinal recovery and reduce nausea within ERAS^®^ [8]. We aimed to compare TEA and CWI for postoperative recovery after open RC within a mature ERAS^®^ program.

## Materials and methods

### Patients and data selection

All consecutive patients undergoing oncological open RC with PLND and urinary diversion at our Swiss tertiary referral center between November 2012 and March 2024 were included. All urinary diversion techniques other than ileal conduit and neobladder were excluded (ureterocutaneostomy: *n* = 19, continent cutaneous diversion: *n* = 12) consistent with the original dataset. Analgesic strategy allocation followed an institutional policy transition: TEA was routine from November 2012 through May 2016, after which subfacial CWI became standard (June 2016-March 2024). No additional study-specific exclusion criteria for CWI were applied beyond the overall cohort eligibility criteria. Groups were therefore chronologically separated in a before-after design. The ethics committee approved the study (CER-VD 2016–02204). All patients provided written informed consent. Demographic, surgical, and postoperative data were captured by a dedicated study nurse through a centralized database hosted on secure servers by Amazon Web Services, Inc. (AWS) in Frankfurt am Main, Germany. The database was accessed via the ERAS^®^ interactive audit system (EIAS) web application. The EIAS export used for this analysis contained complete entries for the primary endpoints. Any secondary missing fields were rare and handled by complete-case analysis without imputation. Postoperative complications were classified using the Clavien–Dindo classification within 90 days [9]. Postoperative pain was assessed using the Visual Analogue Scale (VAS), ranging from 0 (no pain) to 10 (the most severe pain). Due to internal limitations of the EIAS reporting system, only the maximum daily pain intensity was recorded in the database, and it was assumed to occur during mobilization. These daily maxima were used to calculate VAS scores for postoperative days (POD) 1, 2, and 3. Nausea was evaluated using a similar 0 to 10 scale at the same intervals. Gastrointestinal recovery was assessed by time to first flatus, time to first stool, and time to “tolerance of solid food” as a pragmatic, ERAS-relevant surrogate.

### ERAS^®^ protocol

All patients were managed within a standardized ERAS^®^ pathway as previously published [5, 10]. Patients scheduled for RC underwent preoperative counseling by a dedicated nurse. Screening for preoperative anemia was conducted following our institution’s patient blood management policy, based on the three-pillar matrix [11]. Preoperative sedative medications were avoided. Patients received a carbohydrate-rich drink two hours before surgery. Antibiotic prophylaxis was administered 30 min prior to skin incision. Early mobilization was emphasized, beginning on postoperative day (POD) 1. Likewise, early oral feeding was promoted, starting with freely available water 4 h after surgery, with daily progression to normal diet by POD 5. Gut motility was stimulated using chewing gum. Surgical drains were removed early, after excluding urinary leak. For study purposes, adequate oral pain control was defined as achieving a VAS score of less than or equal to 4.

### Anesthesia and analgesia

General anesthesia was maintained with sevoflurane or propofol and fentanyl. Bispectral Index and neuromuscular monitoring were part of standard monitoring equipment. Intraoperative analgesia consisted of intermittent epidural boluses during TEA era and intermittent intravenous fentanyl boluses thereafter. Nausea prophylaxis included dexamethasone and droperidol at induction, and ondansetron at closure. Goal-directed fluid therapy targeted normovolemia [12].

### TEA placement

TEA was placed at the lower thoracic level (Th7-Th10) and verified by test dose. Intraoperative boluses of bupivacaine 0.5% were administered at anesthesiologist discretion. Postoperatively, a continuous epidural infusion was administered, containing low-dose bupivacaine 0.0625%, fentanyl 2 mcg/mL, and epinephrine 2 mcg/mL using patient-controlled epidural analgesia (PCEA) at an infusion rate of 6–12 mL/h with patient-initiated boluses. Paracetamol (1 g q6h) and metamizole (500 mg q6h) were concomitantly administered. Systemic opioids were available as rescue when VAS > 4 despite PCEA. Epidural catheters were typically removed by POD 3–5 after daily assessment by dedicated nurses.

### CWI placement and rescue analgesia

After fascial closure, two multiperforated 12.5 cm incisional catheters (ON-Q^®^ Soaker Catheter™, B. Braun Medical, Sempach, Switzerland) were inserted beneath the rectus sheaths and connected to an elastomeric pump. An initial 10 mL bolus of 0.2% ropivacaine (Sintetica SA, Mendrisio, Switzerland) was administered through each catheter, based on previous studies [8], followed by a continuous infusion at 2 mL/h per catheter (4mL/h in total) for 72 h. The total infused volume was approximately 310 mL, corresponding to a total ropivacaine dose of approximately 620 mg. Paracetamol (1 g q6h) and metamizole (500 mg q6h) were also concomitantly administered. Rescue systemic analgesia consisted of subcutaneous hydromorphone boluses when VAS > 4. Ropivacaine was selected for the present study due to its long-acting kinetics (≈ 6 h; prolonged absorption in peripheral blockade), sensory-selective block, and a safer cardiotoxicity profile than bupivacaine [13]. 

#### Statistical Analysis

Categorical variables were expressed as counts (percentages) and continuous variable were expressed as median (IQR) or means with 95% CI, as appropriate. Between-group comparisons used chi-squared tests for categorical data and either Mann-Whitney U or two-sample *t*-test for continuous data, according to distributional assumptions. Outcomes recorded daily from POD 0–3 (VAS pain, nausea scores, and any opioid rescue) were analyzed with patient-level mixed-effects models to account for within-patient correlation. Linear mixed-effects models were used for VAS pain and nausea, and a mixed-effects logistic model for opioid rescue. Fixed effects included treatment (CWI versus TEA), POD (categorical), and the treatment×POD interaction. All models adjusted for the following prespecified covariates: age, sex, BMI, diabetes, smoking status, ASA class, urinary diversion type, pT, pN, operative time, estimated blood loss, and total intraoperative IV fluids. For continuous outcomes, we report adjusted POD-specific contrasts coded as CWI-TEA (positive values indicate higher scores with CWI). For opioid rescue, odds ratios (ORs) > 1 indicate greater odds with CWI. Model-based 95% CIs are provided for all estimates.

Time to tolerance of solid food and LOS were first compared unadjusted (Mann–Whitney U) and then modeled using multivariable linear regression with robust standard errors, adjusting for the same prespecified covariates. First flatus and first stool were summarized descriptively. Because the analgesic strategy changed by institutional policy with no temporal overlap between groups, treatment assignment was perfectly collinear with calendar time. Therefore, calendar time was not included in adjusted models. Clavien–Dindo complication grade was considered only in a sensitivity analysis due to its potential position on the causal pathway. All tests were two-sided with α = 0.05. Analyses were conducted in Stata^®^ 17 (StataCorp, College Station, TX, USA).

## Results

### Demographic and perioperative data

A total of 242 patients were included (CWI 154 [64%]; TEA 88 [36%]). Baseline demographics were broadly similar (age, sex, diabetes), whereas smoking and pN0 status were more frequent in the CWI group. The TEA group had greater estimated blood loss and higher intraoperative fluid volumes. Operative time and urinary diversion type were comparable between groups. (Table 1).

**Table 1:** Patients’ characteristics of the two studied populations: continuous wound infiltration (CWI) and with thoracic epidural analgesia (TEA)

	CWI	TEA	*p*-value
*N*	154	88	
Age - median (IQR)	70 (63–76)	70.5 (64.5–77)	0.51
Sex-n (%)			0.15
Female	33 (21.4)	26 (29.5)	
Male	121 (78.6)	62 (70.5)	
Smoking - n (%)	88 (57)	33 (37.5)	0.03
Diabetes - n (%)	27 (17.5)	20 (22.7)	0.32
Diversion - n (%)			0.3
Ileal conduit	112 (72.7)	72 (81.2)	
Orthotopic neobladder	42 (27.3)	16 (18.8)	
pT stage (%)			0.42
pT1	23 (14.9)	15 (17.1)	
pT2	40 (26)	13 (14.7)	
pT3	50 (32.4)	31 (35.2)	
pT4	20 (13)	17 (19.3)	
pTis	21 (13.7)	12 (13.7)	
pN stage - n (%)			0.02
pN0	122 (79.2)	48 (54.5)	
pN1	9 (5.8)	16 (18.2)	
pN2	13 (8.4)	17 (19.3)	
pN3	3 (2)	0	
pNx	7 (4.6)	7 (9)	
ASA score - n (%)			0.24
ASA I	0	1 (1.1)	
ASA II	90 (58.4)	55 (62.5)	
ASA III	54 (41.6)	31 (35.3)	
ASA IV	0	1 (1.1)	
Complications-n (%)			0.01
Clavien I	14 (9.1)	4 (4.5)	
Clavien II	65 (42.2)	49 (55.7)	
Clavien IIIa	20 (13.0)	15 (17.1)	
Clavien IIIb	16 (10.4)	14 (15.9)	
Clavien IV*	3 (1.9)	4 (4.5)	
Clavien V	4 (2.6)	2 (2.3)	
No complication	32 (20.8)	0	
OP time - median (IQR)	388 (347–459)	387 (327–438)	0.14
Blood loss - median (IQR)	450(300–600)	800 (500–1200)	< 0.001
IV total ml - median (IQR)	3000 (2500–4200)	4175 (3270–5635)	< 0.001

* IVa and IVb combined

### Postoperative pain, nausea, and opioid rescue

Across POD 0–3, adjusted VAS pain scores were higher with CWI (i.e., TEA provided better early analgesia). POD-specific adjusted contrasts (CWI–TEA) were: POD 0, + 1.90; POD 1, + 0.80; POD 2, + 0.80; POD 3, + 0.90 (all statistically significant; see Table 2 for 95% CIs). Nausea scores were lower with CWI at all time points (e.g., POD 2 contrast − 2.40), with a consistent pattern on POD 0–1 and POD 3 (Table 2). Opioid rescue was more frequent with CWI early after surgery (POD 0 OR 47.40; POD 1 OR 27.00; POD 2 OR 7.36), with differences attenuating by POD 3 (OR 1.57, not significant).

**Table 2:** Model-based contrast by postoperative day (POD) for CWI versus TEA after adjustment

A. Pain - maximum daily VAS during mobilization				
POD	Adjusted mean difference (CWI-TEA)			95% CI
0	+ 1.9*			+ 1.10; +2.70
1	+ 0.8*			+ 0.08; +1.52
2	+ 0.8*			+ 0.13; +1.47
3	+ 0.9**			+ 0.26; +1.54

B. Nausea				
POD	Adjusted mean difference (CWI-TEA)			95% CI
0	-1.2**			-2.0; -0.4
1	-1.7***			-2.5; -0.9
2	-2.4***			-3.34; -1.46
3	-1.5***			-2.34; -0.66

C. Receipt of opioid rescue (yes/no)				
POD	Odds ratio (95% CI)			Risk difference (CWI-TEA) (95% CI)
0	47.4 (9.2–244.7)***			+ 0.44 (0.35; 0.52)
1	27 (7.4–98.9)***			+ 0.41 (0.33; 0.50)
2	7.4 (3.11–17.45)***			+ 0.29 (0.21; 0.39)
3	1.6 (0.81–3.1)			+ 0.08 (-0.03; 0.18)

Panel A: Maximum daily pain on mobilization (VAS 0–10); values are adjusted mean differences (CWI-TEA) with 95% CIs – positive value indicates higher pain with CWI. Panel B: Nausea scores (0–10); values are adjusted mean differences (CWI-TEA) with 95% CIs – negative values indicate less nausea with CWI. Panel C: receipt of opioid rescue (yes/no); values are adjusted odds ratios (ORs) and absolute risk difference (RD = CWI-TEA) with 95% CIs. Models: Patient-level mixed-effects (linear for VAS and nausea; logistic for opioid rescue) with a random intercept for patient; fixed effects for treatment (CWI vs. TEA), POD (0–3), and treatment×POD; adjusted for prespecified covariates. Asterisks denote two-sided significance from the model-based contrasts: *p < 0.05, **p < 0.01, ***p < 0.001

### Gastrointestinal recovery and length of stay

CWI was associated with faster gastrointestinal recovery and shorter hospitalization. In adjusted linear regression, time to tolerance of solid food occurred earlier with CWI (β = −4.4 days; 95% CI, − 6.8 to − 2.1), and LOS was shorter (β = −5.0 days; 95% CI, − 9.1 to − 1.0). First stool occurred earlier with CWI (median 5 vs. 6 days), whereas first flatus showed a nonsignificant trend. Multivariable models are shown in Table 3 (tolerance to solid food) and Table 4 (LOS).

**Table 3:** Univariate and multivariate analyses predicting time to tolerate solid food after open RC

	Univariate analysis						Multivariate analysis				
	Coeff.	Std. Err.	t-value	95% CI [lower; upper]	*p*-value		Coeff.	Std. Err.	t-value	95% CI [lower; upper]	*p*-value
Age	-0.02	0.06	-0.36	[-0.14; 0.11]	0.719						
Gender	-0.73	1.32	-0.55	[-3.34; 1.88]	0.58						
BMI	0.009	0.12	0.88	[-0.23; 0.245]	0.94						
Smoking status	-0.73	1.13	-0.64	[-2.97; 1.51]	0.52						
Length of operation	0.005	0.006	0.84	[-0.007; 0.02]	0.42						
IV total volume	0.006	0.003	1.76	[-0.0008; 0.001]	0.08						
Type of derivation	1.36	1.34	1.02	[-1.28; 3.994]	0.31						
Diabetes	2.43	1.43	1.7	[-0.38; 5.25]	0.09						
Blood loss	0.004	0.00094	4.18	[-0.002; 0.005]	< 0.001		0.002	0.001	1.65	[-0.002; 0.0034]	0.99
ASA Classification	1.624	1.12	1.45	[-0.58; 3.83]	0.04		1.027	1.05	0.97	[-1.05; 3.11]	0.33
Clavien-Dindo score	2.21	0.37	5.9	[-1.47; 2.95]	< 0.001		1.63	0.39	4.17	[0.86; 2.40]	< 0.001
Analgesia (CWI vs. TEA)	-6.39	1.1	-5.76	[-8.57; -4.2]	< 0.0001		-4.42	1.19	-3.72	[-6.76; -2.08]	< 0.0001

**Table 4:** Univariate and multivariate analyses predicting length of stay (LOS)

	Univariate analysis						Multivariate analysis				
	Coeff.	Std. Err.	t-value	95% CI [lower; upper]	*p*-value		Coeff.	Std. Err.	t-value	95% CI [lower; upper]	*p*-value
Age	0.09	0.11	0.87	[-0.12; 0.31]	0.385						
Gender	-2.92	2.41	-1.21	[-7.63; 1.81]	0.226						
BMI	-0.05	0.22	-0.21	[-0.47; 0.28]	0.218						
Smoking status	-0.06	2.06	-0.03	[-4.13; 4.02]	0.907						
Length of operation	0.01	0.02	0.82	[-0.01; 0.03]	0.413						
IV total volume	0.01	0.01	0.66	[-0.009; 0.001]	0.509						
Type of derivation	0.34	2.42	0.14	[-4.43; 5.11]	0.889						
Diabetes	5.74	2.58	2.22	[0.65; 10.82]	0.02		3.76	2.34	1.61	[-0.85; -0.96]	0.109
Blood loss	0.01	0.02	2.73	[0.01; 0.08]	0.007		0.01	0.02	0.26	[0.01; 0.02]	0.794
ASA Classification	5.16	2.01	2.56	[1.2; 9.13]	0.01		2.81	1.87	1.51	[-0.86; 6.49]	0.133
Clavien-Dindo score	5.31	0.62	8.52	[4.09; 6.54]	< 0.0001		4.77	0.66	7.19	[3.46; 6.07]	< 0.001
Analgesia (CWI vs. TEA)	-8.67	2.07	-4.18	[-12.7; -4.6]	< 0.0001		-5.04	2.07	-2.43	[-9.12; -0.96]	0.016

### Complications

Within 90 days, the TEA group experienced more Clavien-Dindo grade II events and slightly more grade IV events. Overall infectious complications were similar between groups. In the CWI group, 20.8% of patients had no recorded complications, and no device-related complications associated with CWI catheter placement were observed.

## Discussion

The assessment of postoperative analgesia commonly relies on pain score and opioid use. However, additional recovery metrics – particularly time to bowel function and LOS, which are intricately linked to postoperative ileus – should also be considered when evaluating analgesic protocols. To date, no single technique has achieved an optimal balance of efficacy, minimal adverse effects, cost-effectiveness, and patient satisfaction. TEA has been widely used for RC, but its role within a contemporary ERAS^®^ is increasingly debated in view of cost and procedure-related risks, alongside the availability of alternative devices such as CWI [14]. A recent meta-analysis concluded that wound catheters are associated with reduced morphine consumption, improved pain scores, greater patient satisfaction, and earlier discharge [15]. We provide an assessment of postoperative bowel recovery (time to first flatus, time to first stool, and time to tolerate oral intake). Postoperative ileus remains common after major abdominal surgery and affects up to one quarter of RC patients, with heterogeneous definitions across studies. Its etiology is likely multifactorial (bowel handling, inflammatory and hormonal responses, splanchnic hypoperfusion, longer operative time) [16]. The shorter gastrointestinal recovery observed with CWI may relate to local anesthetic effects on the peritoneum and potential systemic anti-inflammatory properties [17, 18]. 

Although epidural analgesia has long been associated with accelerated bowel function, much of this evidence derives from comparisons with systemic opioid-based regimens. TEA-related adverse effects—such as hypotension due to sympathectomy, greater fluid requirements, motor block, and intestinal wall edema—may hinder early mobilization and contribute to dizziness and nausea, potentially delaying recovery after cystectomy [7, 19, 20]. In our cohort, opioid use was higher in the CWI group, yet this did not translate into worse bowel recovery or higher nausea scores, suggesting that the between-group difference in opioid exposure had limited clinical impact. Blood-pressure trends would have helped test hypotension-related mechanisms; these were not consistently available in our dataset. Because LOS reflects a composite of analgesia quality, mobilization, gastrointestinal function, and complications, we considered it a key recovery endpoint. Despite superior early pain control with TEA, mobilization was faster with the CWI cohort, with earlier bowel recovery and a shorter LOS. Differences in case-mix may also have contributed. The TEA era had more moderate-to-severe complications and a higher proportion of node-positive disease, both of which plausibly prolong recovery and discharge. In addition, cumulative experience and personnel education over time could have favored the later CWI period in this before-after design [21]. Finally, the marked reduction in LOS observed with CWI implies a potential decrease in hospitalization costs. Although we did not conduct a direct economic analysis, prior studies suggest that CWI can be more cost-effective than TEA for postoperative pain management [22, 23]. Nevertheless, this study is subject to era bias and baseline imbalances (higher smoking and pN0 in CWI; greater blood loss and intraoperative fluids in TEA), and some perioperative variables were incompletely captured (e.g., transfusions, vasopressors, standardized opioid dosing). Infectious complications were captured overall, but wound-specific infection was not coded as a standalone variable, precluding a reliable comparison of surgical site infection rates between groups. To limit confounding, both groups were managed within the same standardized and consistent ERAS^®^ pathway. We used mixed-effects models for repeated outcomes with prespecified covariate adjustment. Importantly, the advantages observed with CWI for recovery endpoints persisted after adjustment – time to tolerance of solid food (adjusted β -4.4 days [95% CI -6.8;-2.1]) and LOS (adjusted β -5.0 days [95% CI -9.1;-1.0]). Pain ascertainment was limited to the daily maximum during mobilization, which may overestimate early pain but applied equally to both groups and aligns with ERAS^®^-relevant activity. Finally, while our LOS exceeds international benchmarks [24], this likely reflects local discharge/rehabilitation practices and complication burden rather than a bias favoring one analgesic strategy.

## Conclusions

Within a mature ERAS^®^ program, CWI emerges as a pragmatic and attractive alternative to TEA after open RC. In our cohort, CWI was associated with faster gastrointestinal recovery, shorter hospital stay, with no device-related complications observed, at the modest trade-off of slightly higher early pain and more frequent early opioid rescue. While these findings stem from a single-center before-after design and should be interpreted considering era-related confounding and local discharge practices, they support broader consideration of CWI in ERAS-guided care for open cystectomy.

## Data Availability

The datasets used and/or analyzed during the current study are available from the corresponding author on reasonable request.
